# Infiltration and sealing for managing non-cavitated proximal lesions: a systematic review and meta-analysis

**DOI:** 10.1186/s12903-020-01364-4

**Published:** 2021-01-07

**Authors:** Yuanyuan Chen, Dongru Chen, Huancai Lin

**Affiliations:** 1 Hospital of Stomatology, Guangdong Provincial Key Laboratory of Stomatology, Sun Yat-Sen University, Guangzhou, China; 2 Department of Orthodontics, Hospital of Stomatology, Guanghua School of Stomatology, Sun Yat-Sen University, Guangzhou, China; 3 Department of Preventive Dentistry, Hospital of Stomatology, Guanghua School of Stomatology, Sun Yat-Sen University, Guangzhou, China; 4 Guangdong Key Laboratory for Dental Disease Prevention and Control, Sun Yat-Sen University, Guangzhou, China

**Keywords:** Infiltration, Sealing, Non-cavitated proximal lesions

## Abstract

**Background:**

Infiltration and sealing are micro-invasive treatments for arresting proximal non-cavitated caries lesions; however, their efficacies under different conditions remain unknown. This systematic review and meta-analysis aimed to evaluate the caries-arresting effectiveness of infiltration and sealing and to further analyse their efficacies across different dentition types and caries risk levels.

**Methods:**

Six electronic databases were searched for published literature, and references were manually searched. Split-mouth randomised controlled trials (RCTs) to compare the effectiveness between infiltration/sealing and non-invasive treatments in proximal lesions were included. The primary outcome was obtained from radiographical readings.

**Results:**

In total, 1033 citations were identified, and 17 RCTs (22 articles) were included. Infiltration and sealing reduced the odds of lesion progression (infiltration vs. non-invasive: OR = 0.21, 95% CI 0.15–0.30; sealing vs. placebo: OR = 0.27, 95% CI 0.18–0.42). For both the primary and permanent dentitions, infiltration and sealing were more effective than non-invasive treatments (primary dentition: OR = 0.30, 95% CI 0.20–0.45; permanent dentition: OR = 0.20, 95% CI 0.14–0.28). The overall effects of infiltration and sealing were significantly different from the control effects based on different caries risk levels (OR = 0.20, 95% CI 0.14–0.28). Except for caries risk at moderate levels (moderate risk: OR = 0.32, 95% CI 0.01–8.27), there were significant differences between micro-invasive and non-invasive treatments (low risk: OR = 0.24, 95% CI 0.08–0.72; low to moderate risk: OR = 0.38, 95% CI 0.18–0.81; moderate to high risk: OR = 0.17, 95% CI 0.10–0.29; and high risk: OR = 0.14, 95% CI 0.07–0.28). Except for caries risk at moderate levels (moderate risk: OR = 0.32, 95% CI 0.01–8.27), infiltration was superior (low risk: OR = 0.24, 95% CI 0.08–0.72; low to moderate risk: OR = 0.38, 95% CI 0.18–0.81; moderate to high risk: OR = 0.20, 95% CI 0.10–0.39; and high risk: OR = 0.14, 95% CI 0.05–0.37).

**Conclusion:**

Infiltration and sealing were more efficacious than non-invasive treatments for halting non-cavitated proximal lesions.

## Introduction

Dental caries is one of the most prevalent oral diseases worldwide [1]. In terms of the susceptibility of the tooth surface to cavitation, the proximal zones have a high risk of being carious [2]. Early proximal caries lesions are prevalent but difficult to observe. Traditionally, invasive treatment methods (drill and fill) have been applied; however, these methods require the removal of marginal tissue and can weaken the strength of the residual tooth structure [3]. In recent years, non-invasive or micro-invasive treatments have been developed to replace traditional restorative treatments. These treatment protocols aim to restore the sound structure in a more preventive way, reduce associated pain and costs, and regain function and aesthetics [4–7]

Non-invasive treatments manage caries lesions via mechanical removal of the biofilm, dietary control or remineralisation treatments [8]. Removal of the biofilm, such as by toothbrushing and interdental flossing, together with dietary control, focused on prevention rather than halting carious lesions [8, 9]. Remineralisation of the enamel lesion with fluoride and casein phosphopeptide amorphous calcium phosphate (CPP-ACP) is promising [7, 10, 11], but it lacks validity without good compliance [7, 12]. Consequently, micro-invasive treatments have been developed as alternatives since they are less dependent upon patient compliance and are more conservative than invasive treatments.

Micro-invasive treatments are applied to manage the lesions confined to the outer third of dentin. They involve the preliminary treatment of the tooth surface. Operators frequently use a conditioning step via organic acid, and micrometres of the enamel layer are removed [13, 14]. The intact surface of the carious lesions is preserved.

Infiltration and sealing are frequently used as micro-invasive treatments. Recently, infiltration technology has been performed clinically for non-cavitated proximal caries [15, 16]. This technique uses low-viscosity resin to occlude the micropores of non-cavitated proximal carious lesions [16, 17]. Based on the capillary force, resin penetrates into the pores of demineralized enamel and establishes a barrier to impede acid diffusion [18, 19]. Thus, micro-porosities are filled, and light scattering of the lesions turns out to be similar to the sound enamel [12]. At the same time, sealing has been investigated to efficiently arrest lesion progression in vivo and in vitro [20–22]. The procedure of sealing involves the application of a resin sealant, glass ionomer cement (GIC), polyurethane tape or adhesives after tooth separation [23–28]. Operators use acid to increase the roughness and afterwards increase the micro-mechanical retention. Resin-based and GIC based sealants are the most commonly used today [29]. They can be light cured to form a layer and impede the invasion of bacteria. In addition, compared to the traditional sealants, polyurethane tapes are regarded as more convenient and easier to handle [23, 24].

Previous systematic reviews and meta-analyses have shown that micro-invasive treatments are more effective than non-invasive treatments [3, 13, 15, 30–32]. However, there is still uncertainty about the intervention effects for patients with different dentition types and different caries risk levels since there have not been sufficient cases to reach a conclusion [15]. Generally, caries management with prevention or therapeutic protocols is based on the caries risk [33]. Thus, to assist in a treatment plan, it is meaningful to justify the intervention effects based on different caries risk levels. In addition, the structure of primary teeth is different from that of the permanent teeth. The thinner and less mineralisation of enamel layer, as well as broader contact area, has a greater likelihood for caries in primary dentition. Researchers found that there was a higher risk of failure in primary teeth with conventional restoration treatments [8, 34, 35]. Thus, whether micro-invasive treatments would influence progression, especially in the primary dentition, would be of great importance for future application. Furthermore, the latest trials are needed to obtain sufficient evidence qualitatively and quantitatively. Therefore, in this study, we conducted a systematic review and meta-analysis to evaluate the efficacies of infiltration and sealing on proximal caries lesions and analysed their efficacies based on different dentition types and caries risk levels.

## Methods

This study was conducted according to the PRISMA statement [36, 37]. The protocols of the eligibility criteria, search strategy, data extraction, risk of bias assessment in the included studies, data synthesis and statistical analysis were prepared.

### Eligibility criteria

The eligibility criteria were designed in accordance with the PICOS strategy.

- Population (P): Children, adolescents and adults, with proximal or approximal non-cavitated caries, presumed clinically (visually intact surface) or by radiographs.
- Interventions (I): Infiltration or sealing technology.
- Comparisons (C): The two micro-invasive strategies were compared to each other and against non-invasive treatments (placebo or no treatment).
- Outcomes (O): Lesion progression was assessed by digital radiography via digital subtraction radiography (DSR), pairwise reading or lesion stage.
- Study design (S): Split-mouth randomised controlled trials (RCTs).

Reviews and meta-analyses, in situ studies, in vitro studies, case reports, study protocols, and meeting abstracts were excluded. Articles were excluded if the patients had a mixture of caries risk levels or if they had high and low caries risk without a specific distribution. Only studies with caries risk for most people (more than 80%) were collected for further classification.

### Search

Electronic databases (Cochrane Library, PubMed, Embase, OpenGrey, ProQuest Dissertations & Theses Global, and Web of Science Conference Proceedings Citation Index-Science (CPCI-S)—2000) were searched by Y.C. and D.C. from inception to April 6, 2020. Two authors (Y.C. and D.C.) selected the eligible studies independently, and disagreements were resolved by discussion and consultation with a third person (H.L.). Eligible studies were explored without limitations on publication type, language, year and region. The following terms were used to search the title, abstract, keywords or MeSH terms: “tooth demineralization OR tooth decay OR caries OR lesion” and “seal OR sealant OR sealing OR infiltrate OR infiltration” and “proximal OR approximal” (“Appendix 1”). A manual search was an auxiliary strategy to improve the comprehensiveness of retrieving studies. Studies were imported into EndNote software, version X9. Duplicates were excluded, and the full texts of the eligible retrieved studies were assessed. Data were requested from authors of the original studies if necessary.

### Data extraction

Data extraction was performed and recorded by two calibrated reviewers independently and in duplicate (Y.C. and D.C.), and disputes were settled by discussion. The titles and abstracts of the studies were initially examined to eliminate irrelevant studies, and then the full texts of the retrieved studies were screened to obtain the included studies. The extracted data included study details (first author and year of publication), patient information (age, sample size, sample type, drop-out rate and caries risk), study design, inventions, and outcome data (caries progression).

### Assessment of risk of bias in the included studies

The risk of bias of the included studies was evaluated according to the criteria in the Cochrane Collaboration’s Risk of Bias Tool (RoB 2) [38]. Researchers must answer signalling questions as follows: bias arising from the randomisation process, bias due to deviations from intended interventions, bias due to missing outcome data, bias measurement of the outcome, bias in selection of the reported result. In addition, drop-out rates less than 25% were regarded to have no substantial impacts of the failure based on previous studies [13, 31]. Overall risk of bias judgement is shown: trials with at least 1 item regarded as high risk were identified as having a high risk of bias. Trials with some concerns in 1 or more key domains were identified as having some concerns about risk. Trials with a low risk of bias in all aspects were identified as having a low risk of bias.

### Heterogeneity assessment

We assessed clinical, methodological diversity and statistical heterogeneity according to the Cochrane Handbook [39]. Clinical heterogeneity involves in the differences among populations, interventions and outcomes. Methodological heterogeneity is associated with the study designs and quality of the studies. Statistically heterogeneity was assessed using a Chi^2^ or *I*^2^ test. Only when the studies have clinical and methodological homogeneity are researchers suggested to have assessment based on statistical heterogeneity.

### Summary measures and data synthesis

The meta-analysis was conducted using Stata software, version 16. Effect variables were calculated as odds ratios (ORs) with 95% confidence intervals (95% CIs) for binary data in this research. Meta-regression analysis was conducted to identify the influence of follow-up years on treatment efficacy.

We conducted the meta-analysis with a random-effects model owing to clinical issues and methodological heterogeneity, regardless of the statistical assessment. The τ^2^ was used to assess statistical heterogeneity. Since differences among the invention methods, dentition types and caries risk levels might have affected the outcome data, we individually analysed these factors using subgroup analysis with a random-effects empirical Bayes model.

### Risk of bias across studies

Publication bias should be considered if more than 10 studies with clinical, methodological and statistical homogeneity are included. Egger’s test and Begg’s test can be used to evaluate publication bias.

### Quality of the evidence

The overall quality of the accrued evidence was assessed with the Grading of Recommendations Assessment, Development and Evaluation (GRADE) [40, 41]. According to GRADE, the evidence was graded as high, moderate, low and very low. High quality indicates high reliability of the estimate. Moderate quality indicates that further research would have an effect on the estimate. Low and very low quality indicate that the true effect could differ from the estimate of the effect. Assessment items were risk of bias, inconsistency, indirectness, imprecision and other considerations (publication bias). We could downgrade one or two levels due to serious or very serious risk of the five domains. In this study, the quality of the evidence was evaluated using GRADEpro (online software).

## Results

### Study selection

**Fig. 1:**
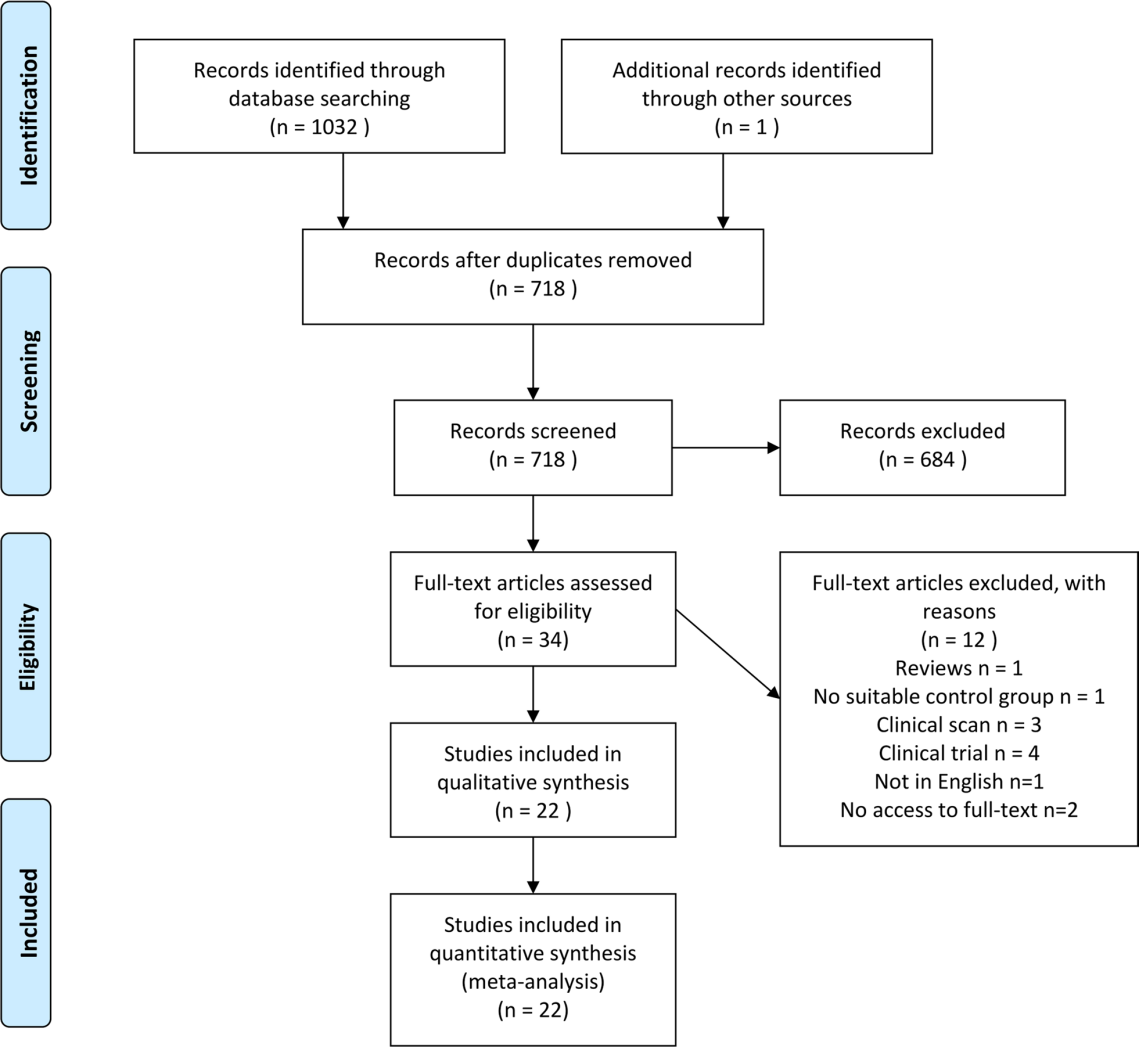
Flow diagram of the study selection. A total of 1033 articles were included, and 22 articles were eligible for quantitative synthesis

A total of 1033 citations were initially identified after an electronic database search (1032 articles) and a manual search (1 article). The selection process was presented as a flow diagram (Fig. 1). Ultimately, 22 articles of the 17 latest studies were included (Tables 1a, b and 2), of which 9 articles were related to 4 different series of studies and 1 article compared infiltration and sealing to the control group individually [23–28, 42–57].

**Table 1:** Characteristics of the included studies

First author (year)	Patient					Study design	Caries risk	Interventions	Control
	Age	Sample size	Lesions treated	Sample type	Drop-out rate				
*(a) Invention: infiltration*									
Ammari [42]	6.2 ± 1.29	50	100	Primary dentition	12 m: 16%, 24 m: 42%	Split-mouth RCT	Moderate to high	Resin infiltration (Icon^®^, DMG, Hamburg, Germany) + fluoridated toothpaste + flossing	Fluoridated toothpaste + flossing
Jorge [48]									
Arslan [43]	20.7 ± 5.65	56	112	Permanent dentition	12 m: 27%	Split-mouth RCT	Moderate to high	Resin infiltration (Icon^®^) + fluoridated toothpaste + flossing	Fluoridated toothpaste + flossing
Arthur [44]	16–41	22	72	Permanent dentition	36 m: 23%	Split-mouth RCT	Unclear	Resin infiltration (Icon^®^) + oral hygiene instruction + dietary advice + topical application of fluoride	Placebo treatment (water) + oral hygiene instruction + dietary advice + topical application of fluoride
Bagher [45]	6.82 ± 1.09	45	90	Primary dentition	24 m: 44%	Split-mouth RCT	Low or high	Resin infiltration (Icon^®^) + fluoride application + oral hygiene + diet counselling	Fluoride application + oral hygiene + diet counselling
Ekstrand [46]	7.17 ± 0.6	48	96	Primary dentition	12 m: 19%	Split-mouth RCT	Moderate to high	Resin infiltration (Icon^®^) + fluoride vanish + regular examinations + oral hygiene instructions	Fluoride vanish + regular examinations + oral hygiene instructions
Foster Page [47]	8 (6–9)	90	180	Primary dentition	24 m: 23%	Split-mouth RCT	Low to moderate	resin infiltration (Icon^®^) + fluoride vanish	Fluoride vanish
Martignon [50]	21 (16–31)	39	117	Permanent dentition	36 m: 5%	Split-mouth RCT	Mixed	Resin infiltration (Icon^®^) or sealant (Prime Bond NT^®^; Dentsply, York, PA, USA) + flossing	Placebo treatment (micro-brush) + flossing
Meyer-Lueckel [51]	23 ± 6	79	436	Permanent dentition	18 m: 11%	Split-mouth RCT	Low or high	Resin infiltration (Icon^®^) + fluoride vanish + oral hygiene instruction + dietary advice	Mock infiltration + fluoride vanish + oral hygiene instruction + dietary advice
Paris [54]	25 (20–34)	22	58	Permanent dentition	18 m: 0%	Split-mouth RCT	Mixed	Resin infiltration (Icon^®^) + fluoridation + oral hygiene + dietary instructions	Placebo treatment (water) + fluoridation + oral hygiene + dietary instructions
Meyer-Lueckel [52]					36 m: 9%				
Paris [53]					84 m: 27%				
Peters [55]	20.1 ± 0.9	42	84	Permanent dentition	24 m: 24%	Split-mouth RCT	High	Resin infiltration (Icon^®^) + fluoride vanish + hygiene instruction + diet counselling + fluoridated toothpaste	Mock infiltration + fluoride vanish + hygiene instruction + diet counselling + fluoridated toothpaste
Peters [56]					36 m: 36%				
Vaghela [57]	26 (14–45)	56	130	Permanent dentition	6 m: 51.79%	Split-mouth RCT	low or moderate	Resin infiltration (Icon^®^, DMG, Hamburg, Germany) + standard-oral care hygiene treatment + diet counseling + a fluoride regimen	With inactive materials + standard-oral care hygiene treatment + diet counseling + a fluoride regimen
*(b) Intervention: sealing*									
Alkilzy [23]	21.3 ± 5.6	50	100	Permanent dentition	24 m: 30%	Split-mouth RCT	Unclear	Sealant patch (Ivoclar Vivadent, Principality of Liechtenstein) + fluoridated toothpaste + dental floss	Fluoridated toothpaste + dental floss
Alkilzy [24]					36 m: 40%				
Basili [25]	8.5 ± 0.7	25	50	Permanent dentition	42 m: 40%	Split-mouth RCT	High	Sealant (Concise Sealant; 3 M ESPE) + fluoride vanish (Duraphat; Colgate Oral Pharmaceuticals) + general oral hygiene instructions + dietary advices	Fluoride vanish (Duraphat; Colgate Oral Pharmaceuticals) + general oral hygiene instructions + dietary advices
Gomez [26]	14.7 ± 2.1	7	71	Permanent dentition	24 m: 0%	Split-mouth RCT	Unclear	Pit and fissure sealants (Concise sealant; 3 M ESPE)	Fluoride varnish (Duraphat; Colgate Oral Pharmaceuticals, Canton, MA, USA)
Martignon [49]	15–39	82	164	Permanent dentition	18 m: 12%	Split-mouth RCT	Moderate to high	Sealant (Gluma One Bond adhesive, Heraeus Kulzer; Concise sealant, 3 M ESPE) + flossing	Flossing
Martignon [27]	5.3 ± 0.7	91	182	Primary dentition	30 m: 38%	Split-mouth RCT	Mixed	Sealant (Single One Bond, 3 M ESPE) + flossing	Flossing
Martignon [50]	21 (16–31)	39	117	Permanent dentition	36 m: 5%	Split-mouth RCT	Mixed	Resin infiltration (Icon^®^) or sealant (Prime Bond NT^®^; Dentsply, York, PA, USA) + flossing	Placebo treatment (micro-brush) + flossing
Trairatvorakul [28]	13.15 ± 3.47	26	82	Permanent dentition	12 m: 0%	Split-mouth RCT	Unclear	Glass ionomer cements (GIC, Fuji VII, GC Corp., Tokyo, Japan) + sodium fluoride dentifrice + acidulated phosphate fluoride gel	Sodium fluoride dentifrice + acidulated phosphate fluoride gel

**Table 2:** Caries progression of included studies

First author (year)	Assessment	Follow-up (months)	Test group		Control group	
			Progression	Total	Progression	Total
Alkilzy [23]	Independent reading	24	2	35	2	35
Alkilzy [24]						
		36	2	30	2	30
Arthur [44]	Pairwise reading	36	2	27	5	27
Arslan [43]	DSR	12	1	45	9	45
Ammari [42]	Pairwise reading	12	5	42	14	42
Jorge [48]						
		24	7	29	16	29
Bagher [45]	Pairwise reading	6	5	44	7	44
		12	6	41	13	41
		18	7	31	13	31
		24	10	25	18	25
Basili [25]	Pairwise reading	42	3	15	8	15
Ekstrand [46]	Independent reading	12	9	39	24	39
Foster Page [47]	Pairwise reading	12	15	66	30	69
Gomez [26]	Independent reading	24	3	38	4	33
Martignon [49]	Independent reading	18	7	72	19	72
	Pairwise reading		16	72	34	72
	DSR		30	69	58	69
Martignon [27]	Independent reading	12	20	73	37	73
		30	26	56	40	56
Martignon [50]	Pairwise reading	12	Infiltration: 6	38	18	38
			Sealing: 11			
		24	Infiltration: 9	37	23	37
			Sealing: 15			
		36	Infiltration: 12	37	26	37
			Sealing: 15			
	DSR	12	Infiltration: 10	38	24	38
			Sealing: 16			
Meyer-Lueckel [51]	Pairwise reading	18	10	186	58	186
Paris [54]	Independent reading	18	1	27	2	27
Meyer-Lueckel [52]						
Paris [53]						
	Pairwise reading	18	1	27	6	27
	DSR	18	2	27	10	27
	Pairwise reading	36	1	26	9	26
	DSR	36	1	26	11	26
	Pairwise reading	84	1	22	9	22
	DSR	84	2	22	10	22
Peters [55]	Independent reading	24	0	34	3	34
Peters [56]						
	Pairwise reading	24	1	34	9	34
	Independent reading	36	3	29	7	29
	Pairwise reading	36	4	29	14	29
Trairatvorakul [28]	Pairwise reading	12	0	41	3	41
Vaghela [57]	Pairwise reading	6	0	30	3	30

### Characteristics of the included studies

The data from included studies were summarised in Tables 1 and 2. All of the studies were split-mouth RCTs. A total of 830 patients (ranging from 4.6 to 45 years old) were enrolled in 17 clinical trials. There were 2124 non-cavitated proximal lesions in the trials. A total of 5 studies were included that assessed lesions in the primary dentition [27, 42, 45–48], and 12 studies assessed lesions in the permanent dentition [23–26, 28, 43, 44, 49–57]. The interventions included resin infiltration (11 studies) [42–48, 50–57] and sealant (7 studies) [23–28, 49, 50]. The follow-up duration ranged from 6 to 84 months. In terms of caries risk levels, 2 studies reported high risk [25, 55, 56], 4 studies reported moderate to high risk [42, 43, 46, 48, 49], 1 study reported low to moderate risk [47], 1 study reported low or moderate risk [57], 2 studies reported low or high risk [45, 51], 3 studies reported mixed risk levels [27, 50, 52–54] and 4 studies did not report caries risk in the articles [23, 24, 26, 28, 44]. Five caries risk statuses were included in the subgroup analysis: low [51, 57], low to moderate [47], moderate [57], moderate to high [42, 43, 46, 48, 49] and high [25, 51, 55, 56]. All of the trials used radiographic lesion progression as the primary outcome. Methods for evaluating lesion progression included independent reading of radiographs, pairwise reading of radiographs and DSR. For data analysis, the most sensitive outcome was recorded if two or more evaluation methods were used in a study (outcomes obtained by DSR > pairwise reading > independent reading).

### Risk of bias within studies

**Fig. 2:**
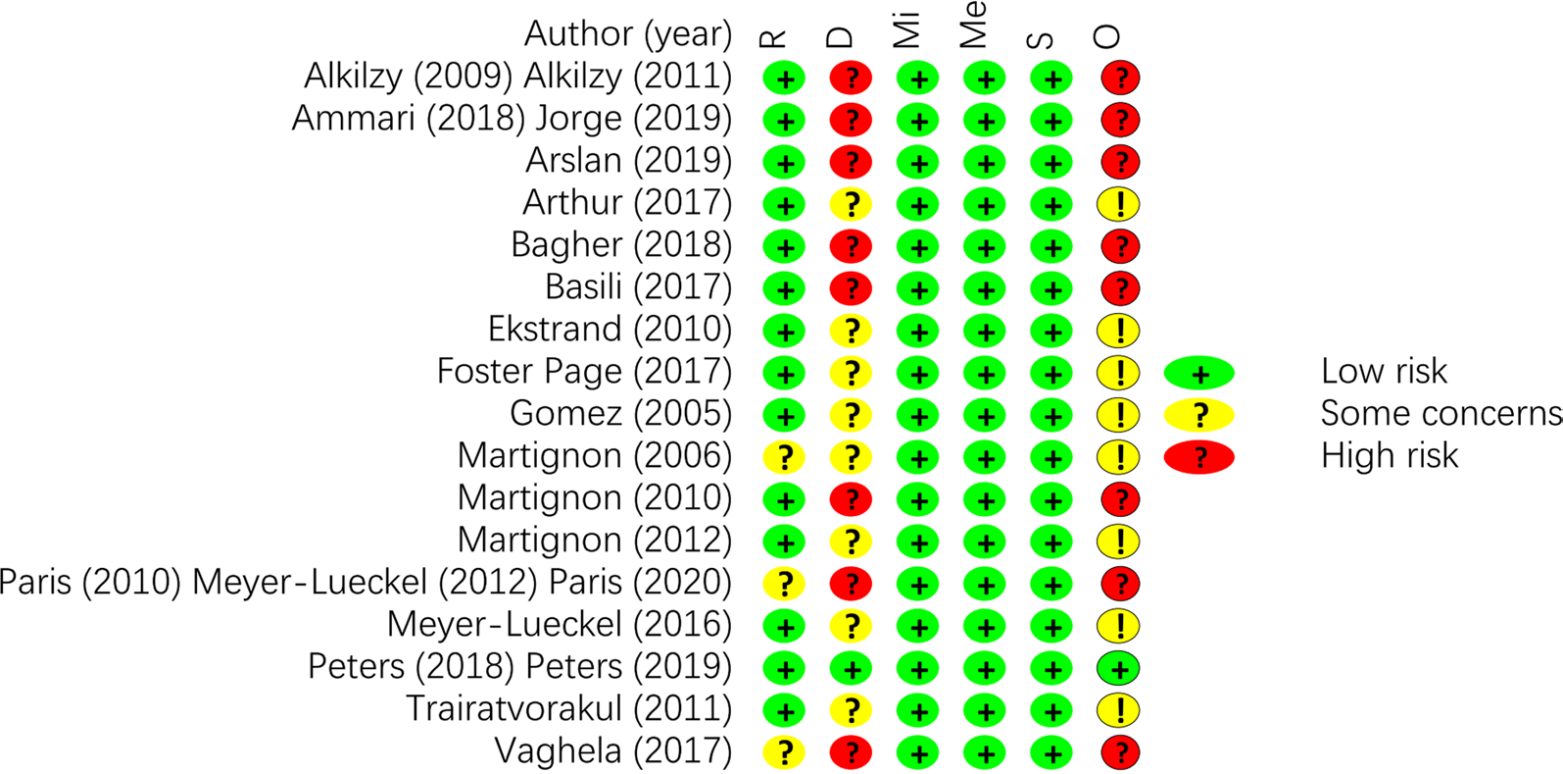
Risk of bias summary of the included studies. In this chart, green circles represent a low risk of bias, yellow circles represent some concerns of bias, and red circles represent a high risk of bias

**Fig. 3:**
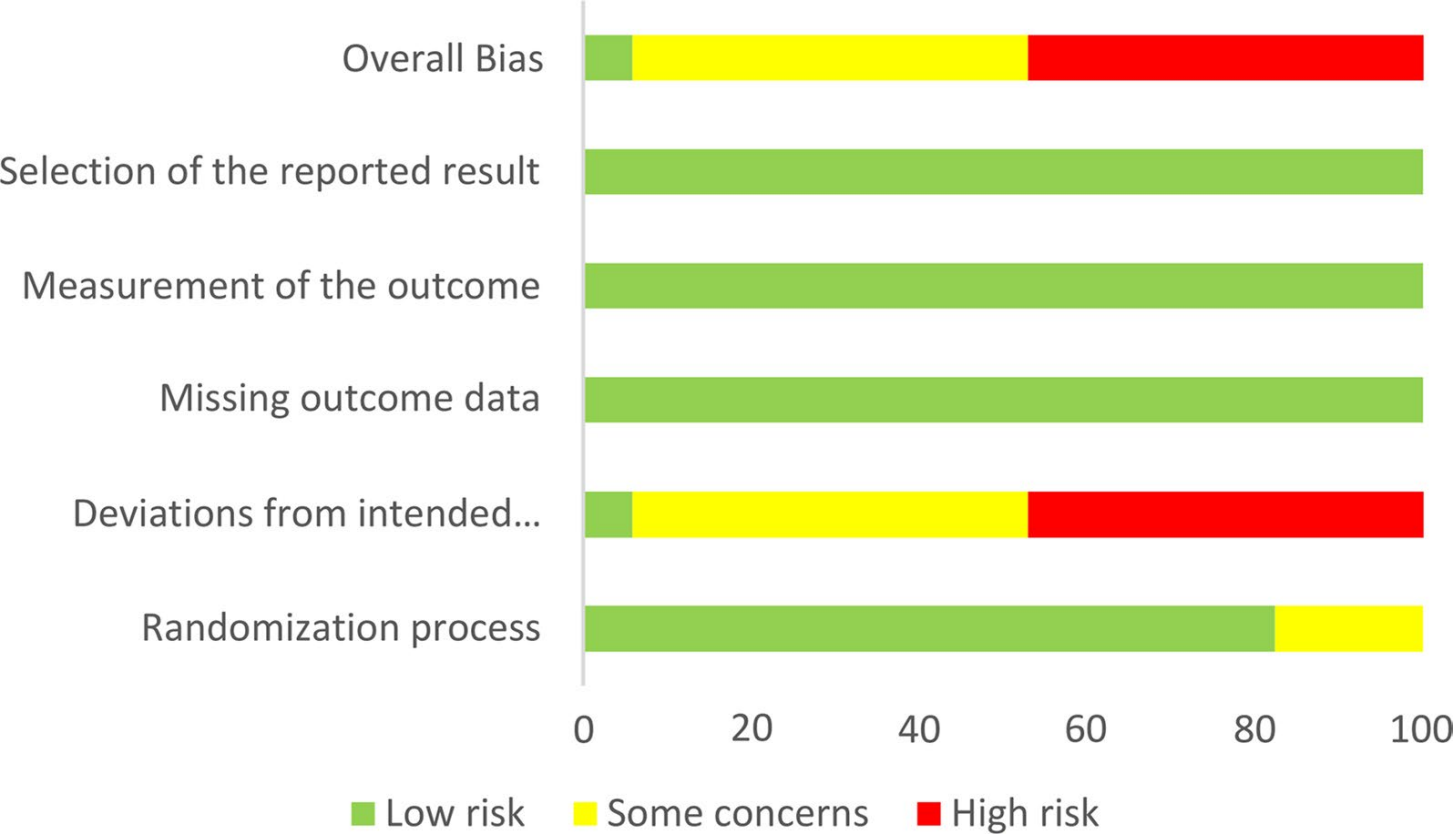
Risk of bias graph. In this graph, green bars represent a low risk of bias, yellow bars represent some concerns of bias, and red bars represent a high risk of bias

The risk of bias within studies was summarised in Figs. 2 and 3. Except for 3 studies with unclear risk for randomisation process due to unbalanced distribution of lesions at baseline [49, 52–54, 57], the remaining studies all had a low risk of bias [23–26, 28, 42–51]. Eight studies had some concerns due to deviations from intended interventions [26, 28, 44, 46, 47, 49–51] while 8 studies have high risk [23–25, 27, 43, 45, 52–54, 57] and 1 study has low risk [55, 56]. All of the studies had low risk for bias due to missing outcome data, measurement of the outcomes and selection of the reported results.

### Heterogeneity assessment

For clinical heterogeneity, sealing and infiltration were two types of invention treatments enrolled as micro-invasive treatments. For non-invasive treatments, it differed across studies. Five studies had placebo treatments, while flossing, fluoride application and dietary advice were also set as comparators. Further, in different studies, these comparators were not combined totally and consistently. Independent reading, pairwise reading, and DSR were used as outcome assessments and varied in studies. In addition, results of bias due to deviations from intended interventions turned out to be due to inconsistency in methodological assessments. No statistical heterogeneity was found between studies (τ^2^ = 0).

### Meta-regression analysis

The meta-regression analysis results revealed that different research durations (ranging from 6 to 84 months) did not influence caries progression (P >|t|: 0.620, 95% CI − 0.143 to 0.233). Thus, we chose caries progression at the longest follow-up times for continuous RCTs, similar to previous reviews [3, 13, 30].

### Efficacy of infiltration and sealing for non-cavitated proximal caries

**Fig. 4:**
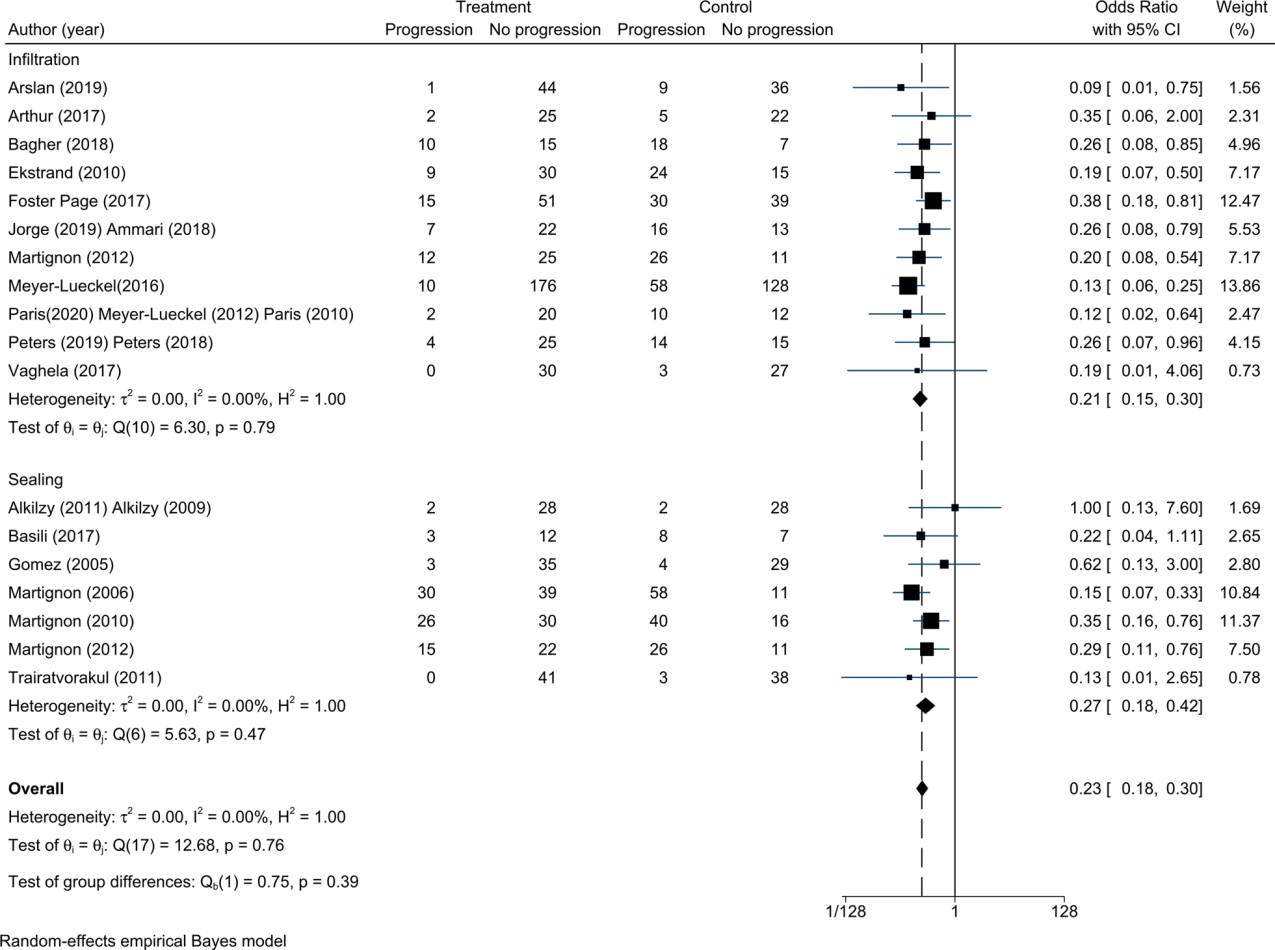
Comparison of the efficacy between infiltration and sealing. The overall effects of infiltration and sealing were significantly different from the control effects (OR = 0.23, 95% CI 0.18–0.30). Both infiltration and sealing were more effective than non-invasive treatments (infiltration vs. non-invasive treatments: OR = 0.21, 95% CI 0.15–0.30; sealing vs. placebo: OR = 0.27, 95% CI 0.18–0.42)

Seventeen RCTs were enrolled to assess the efficacy of infiltration and sealing for non-cavitated proximal caries. A random-effects model was used even though there was no significant statistical heterogeneity between studies (τ^2^ = 0.00, Fig. 4). The overall intervention effects of infiltration and sealing were significantly different from the intervention effects of the control treatment (OR = 0.23, 95% CI 0.18–0.30). We analysed the two different measures (infiltration and sealing) using subgroup analysis, and we found that both invention measures reduced the odds of lesion progression compared with the control group (infiltration vs. non-invasive treatments: OR = 0.21, 95% CI 0.15–0.30; sealing vs. placebo: OR = 0.27, 95% CI 0.18–0.42).

**Fig. 5:**
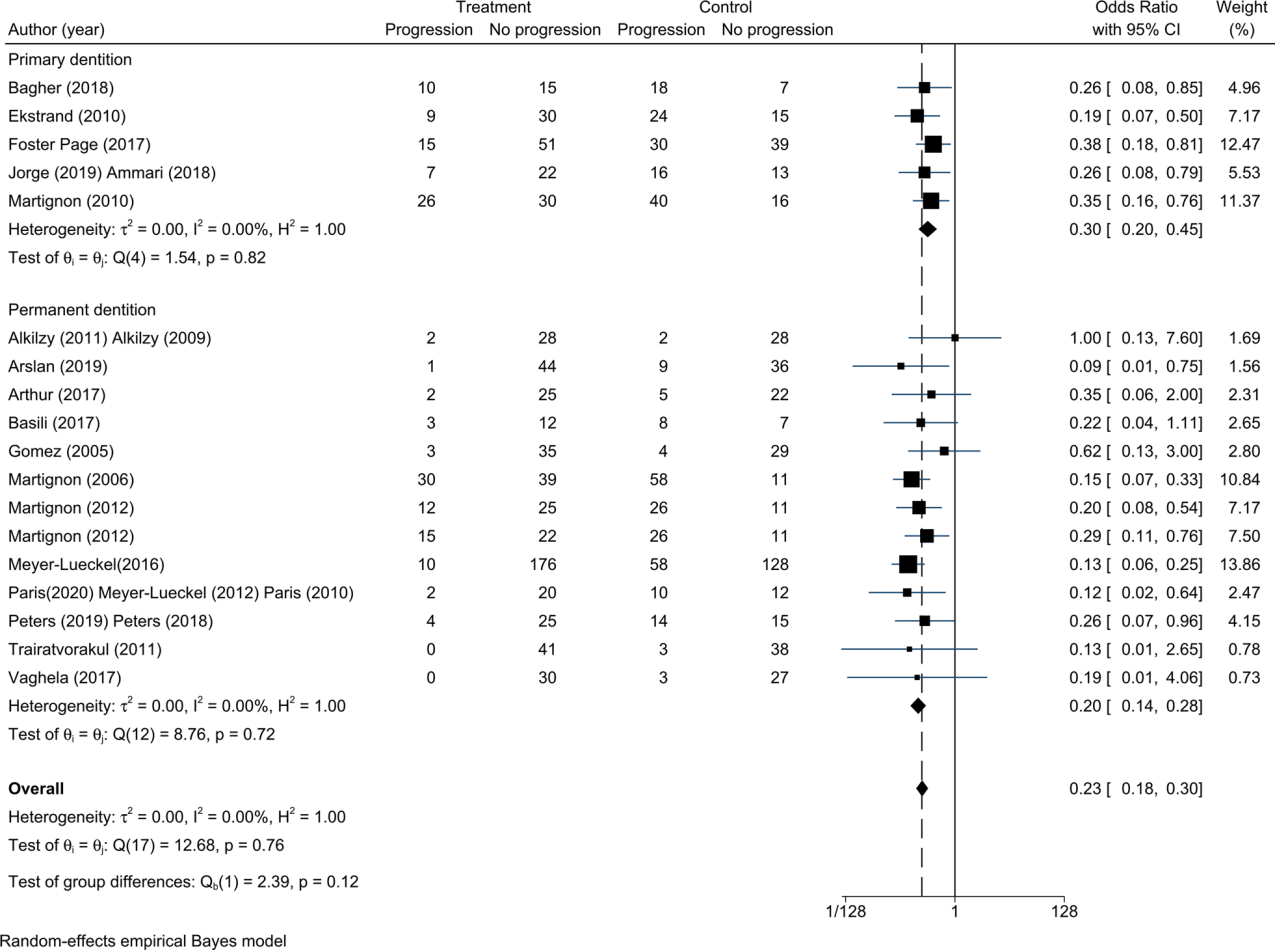
Comparison of the efficacy between primary dentition and permanent dentition. The overall effects of micro-invasive treatments were significantly different from the control effects (OR = 0.23, 95% CI 0.18–0.30). Both infiltration and sealing were more effective than non-invasive treatments in primary dentition and permanent dentition (primary dentition: OR = 0.30, 95% CI 0.20–0.45; permanent dentition: OR = 0.20, 95% CI 0.14–0.28)

Seventeen RCTs were related to infiltration and sealing of primary dentition or permanent dentition. There was no significant statistical heterogeneity of the included RCTs (τ^2^ = 0.00, Fig. 5). Non-cavitated proximal lesions were reduced when measures were undertaken in the primary dentition and permanent dentition (primary dentition: OR = 0.30, 95% CI 0.20–0.45; permanent dentition: OR = 0.20, 95% CI 0.14–0.28, Fig. 5).

**Fig. 6:**
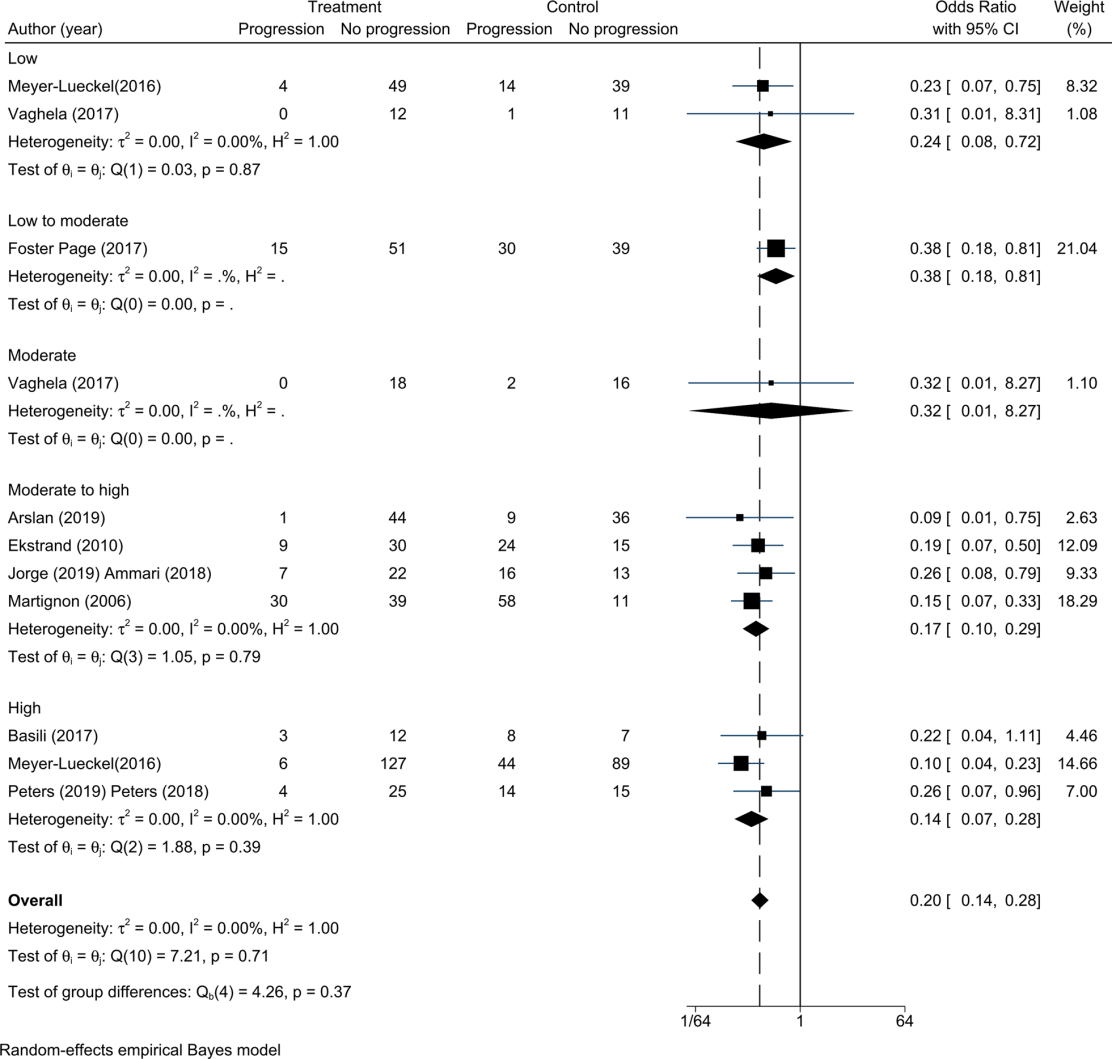
Comparison of the efficacy for different caries risks. Except for patients with moderate risk (OR = 0.32, 95% CI 0.01–8.27), there were significant differences between micro-invasive treatments and non-invasive treatments (low risk: OR = 0.24, 95% CI 0.08–0.72; low to moderate risk: OR = 0.38, 95% CI 0.18–0.81; moderate to high risk: OR = 0.17, 95% CI 0.10–0.29; and high risk: OR = 0.14, 95% CI 0.07–0.28)

**Fig. 7:**
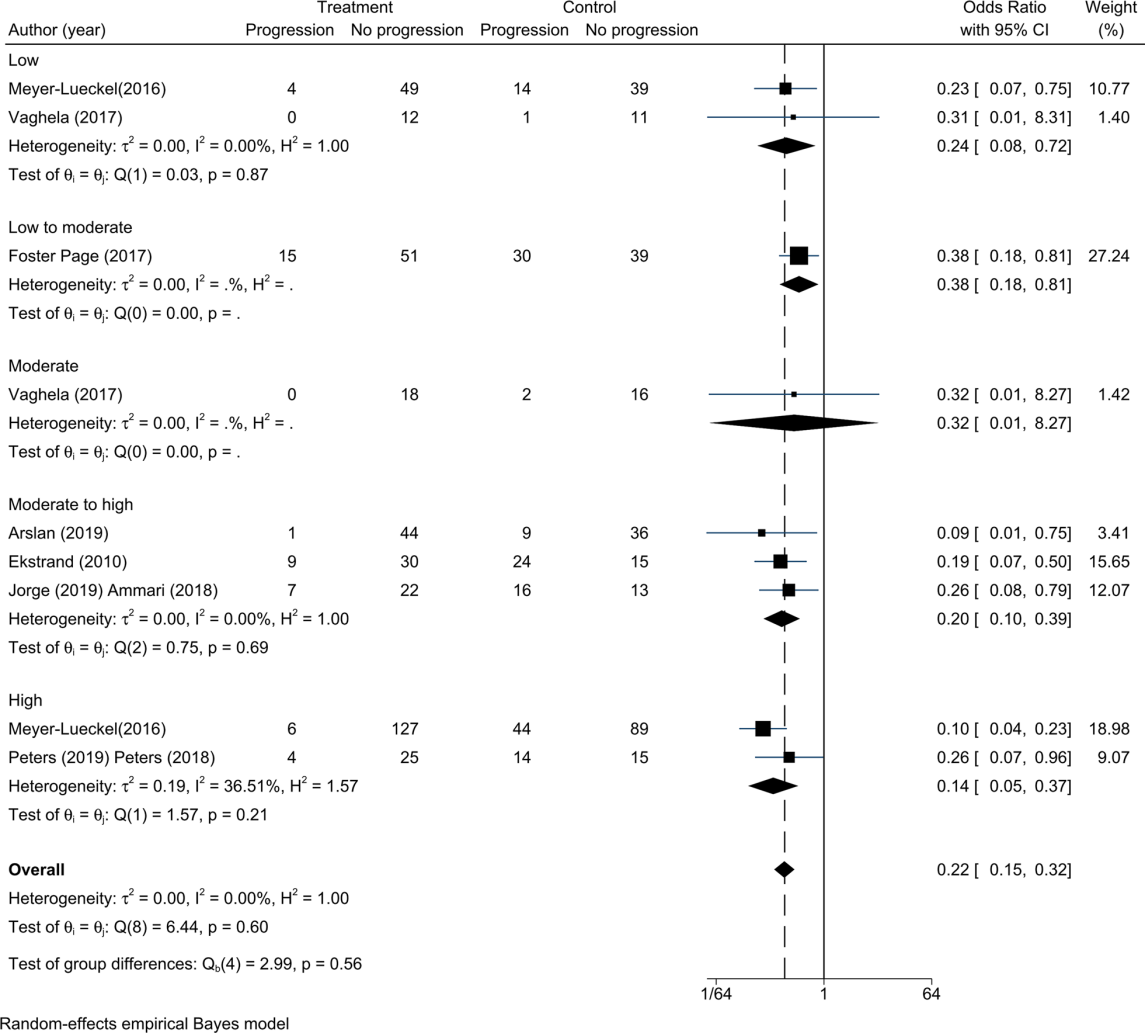
Comparison of the efficacy for different caries risks in infiltration. Except for patients with moderate risk (OR = 0.32, 95% CI 0.01–8.27), infiltration was superior to non-invasive treatments for patients with different caries risk levels (low risk: OR = 0.24, 95% CI 0.08–0.72; low to moderate risk: OR = 0.38, 95% CI 0.18–0.81; moderate to high risk: OR = 0.20, 95% CI 0.10–0.39; and high risk: OR = 0.14, 95% CI 0.05–0.37)

Nine RCTs were analysed for the efficacy of infiltration and sealing at different caries risk levels (Table 1a, b). There was no significant statistical heterogeneity among the nine RCTs (τ^2^ = 0.00, Fig. 6). The overall effects of infiltration and sealing were significantly different from the overall effects of control treatment (OR = 0.20, 95% CI 0.14–0.28). For patients with different caries risk levels, there were significant differences between micro-invasive treatments and non-invasive treatments (low risk: OR = 0.24, 95% CI 0.08–0.72; low to moderate risk: OR = 0.38, 95% CI 0.18–0.81; moderate to high risk: OR = 0.17, 95% CI 0.10–0.29; and high risk: OR = 0.14, 95% CI 0.07–0.28) except for moderate risk: (OR = 0.32, 95% CI 0.01–8.27). Seven RCTs were related to infiltration at different caries risk levels. There was no significant statistical heterogeneity among the seven RCTs (τ^2^ = 0.00, Fig. 7). In contrast to patients with moderate caries risk (OR = 0.32, 95% CI 0.01–8.27), significant differences in the progression rate were found among patients who were treated with infiltration and non-invasive treatments (low risk: OR = 0.24, 95% CI 0.08–0.72; low to moderate risk: OR = 0.38, 95% CI 0.18–0.81; moderate to high risk: OR = 0.20, 95% CI 0.10–0.39; and high risk: OR = 0.14, 95% CI 0.05–0.37). Two RCTs were related to sealing across different caries risk levels. Due to insufficient patient information in terms of caries risk levels in the sealing group, no subgroup analysis was conducted.

### Publication bias

For this meta-analysis, publication bias was not evaluated due to insufficient studies (fewer than 10) with clinical and methodological homogeneity.

### Quality of evidence

Based on this study, infiltration or sealing arrested progression in 283 lesions per 1000 treated lesions. Infiltration arrested progression in 275 lesions per 1000 treated lesions. Sealing arrested progression in 288 lesions per 1000 treated lesions. It was downgraded one level mainly owing to a high risk of bias in half of the included studies. All of the evidence was graded as moderate (“Appendix 2”).

## Discussion

Micro-invasive inventions represent promising approaches for treating proximal lesions. Based on this study, infiltration and sealing can be considered effective micro-invasive inventions for halting the progression of non-cavitated proximal caries. These results were consistent with previous studies [3, 15, 32]. Based on GRADEpro, all of the included studies led to a moderate quality of evidence. We downgraded the quality due to the high risk of bias when evaluating the deviations from intended interventions. In addition, a small proportion of included studies (three studies) had unclear risk for randomisation process due to unbalanced distribution of lesions at baseline, but we did not downgrade the quality again since overall high risk of bias in two studies were already evaluated. As for the inconsistency, there was no statistical heterogeneity between studies; thus we did not downgrade the quality. The publication bias was not evaluated due to a lack of sufficient studies, and we did not downgrade. Therefore, the conclusions from this research are robust and reliable.

With this limited research, our study could not identify a superior micro-invasive treatment for clinical application. Nevertheless, a comparison of infiltration and sealing in terms of clinical procedure could be performed. Infiltration is considered simple and acceptable for patients [42, 47, 58]. After the application of topical anaesthesia to reduce pain and the placement of the wedge, the resin penetrated the proximal lesions, and only one visit was needed for application [32, 47, 55, 56]. Comparatively, sealing is more complex than infiltration since it requires two visits [23–27]. In addition, the commercial product “Icon” is available for standard application in resin infiltration [32]. Thus, with regard to clinical application, infiltration seems to be more suitable. Moreover, a network meta-analysis revealed that infiltration is more likely to be effective than sealing [32]. Conversely, an in vitro study showed that sealing might be more effective in preventing enamel dissolution [59], and the remaining roughness and micro-leakage after infiltration could cause plaque accumulation and biofilm formation [59–63]. Therefore, resolving these disputes requires further trials to directly compare the efficiency, applicability and cost between infiltration and sealing [32].

Based on this research, and according to subgroup analysis, infiltration and sealing are appliable regardless of dentition type. Currently, only one study has concluded that sealing is effective at halting lesion progression both in the primary dentition and the permanent dentition [30]. In other meta-analyses, due to a lack of sufficient data, no robust conclusions could be drawn regarding primary teeth [15]. Although trials for primary teeth seem to be more complicated, and it is more difficult to ensure proper controls, investigations into the efficacy of micro-invasive treatments for primary teeth are necessary and meaningful. Specifically, comfort and acceptability during the treatment of primary teeth are worth evaluating [42, 47]. Furthermore, follow-up times are limited to more than 24 months for primary dentition due to the exfoliation of primary teeth. For 5 studies enrolled in this research, we could conclude that micro-invasive treatments were more effective than non-invasive treatments in the primary dentition for the period from 12 to 24 months. Thus, there are new insights into the treatment of non-cavitated proximal caries in primary teeth since micro-invasive treatments not only reduce children’s pain and fear but also are efficacious. More studies of primary teeth are warranted to reach more reliable conclusions.

To improve efficiency under different clinical conditions, trials are conducted in terms of patients with different caries risk levels. A previous review indicated that the progression rate of non-cavitated proximal lesions was highly relevant to the individual caries risk [64]. Thus, conducting a caries risk assessment beforehand is vital and should be considered a prerequisite. A caries risk assessment would help in caries management and oral care plans [65, 66]. In most of the included studies, caries risk levels were evaluated based on the Cariogram or modified Cariogram. Cariogram is a frequently used multifactorial risk assessment model for individuals [67]. Generally, caries risk ranges from low to high. A high caries risk means greater likelihood of being infected with new caries, a higher frequency for preventive instruction, as well as the application of fluoride, and a higher possibility of needing restoration [68]. Therefore, to elucidate the relationship between the caries risk levels and the efficacy of micro-invasive treatments, we divided the enrolled patients into four groups and then conducted subgroup analyses. Previously, four studies concluded that caries progression was not related to the caries risk levels at baseline [42, 45, 51, 69]. However, one study concluded that, in children with moderate caries risk, lesion progression was 4 times higher than that in children with low caries risk [47]. In addition, one study demonstrated that there was a moderate relationship between increasing caries risk and lesion progression [27]. In this research, it was shown that micro-invasive treatments could effectively halt caries progression at most caries risk levels. Nevertheless, patients with low caries risk are expected to have slower caries progression [47] and to require more preventive treatments, compared to therapeutic protocols to halt caries progression [33, 57]. Non-invasive treatments are regarded as ethical and should be considered part of the treatment plan, especially when the disease process is controlled [44, 70]. However, patients might refuse non-invasive treatments and favour invasive treatments under some circumstances [71]. Thus, for patients with proximal caries lesions, micro-invasive treatments seem to be a meaningful and important choice. In addition, the results of subgroup analysis with the infiltration group showed the same tendency as the results for the overall effect. Therefore, with a limited number of studies, we concluded that micro-invasive treatments could be effective options.

This study showed some strengths that enhance its reliability. To the best of our knowledge, this study was the first to evaluate the efficiency of micro-invasive treatments based on different caries risk levels. In addition, there were more studies in this review than in previously published reviews. All of the studies were RCTs and had a split-mouth design, which helped to improve the validity of the trials. Furthermore, there was no statistically significant heterogeneity among the enrolled studies.

Nevertheless, this review also had some limitations that should be mentioned. First, as a consequence of the limited numbers of studies, patients were divided into rough groups, and each group presented the majority of the caries risk levels in the samples. For further research, it is necessary to determine caries risk levels for every patient and to perform a detailed and precise assessment. Second, the outcome assessment of the included studies varied among independent reading, pairwise reading, and DSR. A standardised method would have been better for outcome evaluation. Otherwise, with a sufficient number of included studies, researchers could conduct subgroup analysis according to the different methods of radiographic assessment, as previously reported [13]. Third, most of the studies had moderate to high risk of bias due to the deviations from intended interventions. One reason was that the blinding of patients is feasible through placebo treatment, yet the blinding of operators is difficult to arrange. The other reason was that most of the included studies were calculated with per-protocol analysis; however, some studies have argued that, in the split-mouth design, it is doubtful whether attrition will affect the overall risk of bias [13, 27]. Thus to qualify the studies, when the drop-out rate was more than 25%, the missing data were regarded to have potential impacts on the results [13, 31]. Finally, the lack of pre-registration of the this study would be of great risk since the same type of meta-analysis would be published repeatedly.

## Conclusions

In summary, infiltration and sealing were more efficacious than non-invasive treatments for arresting the progression of proximal carious lesions. In both the primary and permanent dentition, infiltration and sealing were effective. For the intervention effects of infiltration or sealing on different caries risk levels, a larger number of trials and more detailed trials are needed for further exploration. For future studies, investigations into the efficacy, feasibility and cost-effectiveness of infiltration versus sealing remain necessary.

## Data Availability

All data generated or analysed during this study are included in this published article [and its supplementary information files].

## Appendix 1: Search strategy of databases (search date: 4.6, 2020)

Cochrane trials

**Table:**

#1	tooth demineralization in Title Abstract Keyword OR tooth decay in Title Abstract Keyword OR caries in Title Abstract Keyword OR lesion in Title Abstract Keyword *(Word variations have been searched)*	46252
#2	seal in Title Abstract Keyword OR sealant in Title Abstract Keyword OR sealing in Title Abstract Keyword OR infiltrate in Title Abstract Keyword OR infiltration in Title Abstract Keyword (Word variations have been searched)	16026
#3	proximal in Title Abstract Keyword OR approximal in Title Abstract Keyword	58837
#4	#1 AND #2 AND #3	180

Pubmed

**Table:**

#1	Search ((((tooth demineralization[Title/Abstract]) OR tooth decay[Title/Abstract]) OR caries[Title/Abstract]) OR lesion[Title/Abstract]) OR tooth demineralization[MeSH Terms]	380756
#2	Search ((((sealant[Title/Abstract]) OR seal[Title/Abstract]) OR sealing[Title/Abstract]) OR infiltrate[Title/Abstract]) OR infiltration[Title/Abstract]	169858
#3	Search (((proximal[Title/Abstract]) OR approximal[Title/Abstract])	2095147
#4	Search (((((((tooth demineralization[Title/Abstract]) OR tooth decay[Title/Abstract]) OR caries[Title/Abstract]) OR lesion[Title/Abstract]) OR tooth demineralization[MeSH Terms])) AND (((((sealant[Title/Abstract]) OR seal[Title/Abstract]) OR sealing[Title/Abstract]) OR infiltrate[Title/Abstract]) OR infiltration[Title/Abstract])) AND ((proximal[Title/Abstract]) OR approximal[Title/Abstract])	304

Embase

**Table:**

#1	'caries':ab,ti OR 'tooth decay':ab,ti OR 'lesion':ab,ti OR 'tooth demineralization':ab,ti	503489
#2	proximal:ab,ti OR approximal:ab,ti	275661
#3	seal:ab,ti OR sealing:ab,ti OR sealant:ab,ti OR infiltrate:ab,ti OR infiltration:ab,ti	240444
#4	#1 AND #2 AND #3	510

Open grey

**Table:**

infiltration AND proximal	5
infiltration AND approximal	0
infiltrate AND proximal	1
infiltrate AND approximal	0
seal AND proximal	0
seal AND approximal	0
sealant AND proximal	0
sealant AND approximal	0
sealing AND proximal	0
sealing AND approximal	0
Total	6

ProQuest dissertations & theses global

**Table:**

all(tooth demineralization OR tooth decay OR caries OR lesion) AND all(approximate OR proximal) AND all(seal* OR infiltrat*)	17

Web of Science Conference Proceedings Citation Index-Science (CPCI-S)—2000–10.13 2020

**Table:**

#1	tooth demineralization OR tooth decay OR caries OR lesion	49007
#2	proximal OR approximal	11892
#3	infiltrat* OR seal*	34196
#4	#1 AND #2 AND #3	15

## Appendix 2.1: Interactive SoF of the included studies

Quality of evidence evaluated by GRADEpro (online software)

**Table:**

Outcomes	Plain language statements	Absolute effect		Relative effect (95% CI)	Certainty of the evidence GRADE
		With non-invasive treatments	With micro-invasive treatments		
Measures	Lesions progression after infiltration or sealing	432	149	OR:0.23 (0.18–0.30)	⊕ ⊕ ⊕ ○ moderate
		283 fewer per 1000 patients (95% CI 312–246 fewer per 1000 patients)			
Measures: infiltration	Lesions progression after infiltration	396	121	OR:0.21 (0.15–0.29)	⊕ ⊕ ⊕ ○ moderate
		275 fewer per 1000 patients (95% CI 306–236 fewer per 1000 patients)			
Measures: sealing	Lesions progression after sealing	502	214	OR:0.27 (0.18–0.42)	⊕ ⊕ ⊕ ○ moderate
		288 fewer per 1000 patients (95% CI 348–205 fewer per 1000 patients)			

## Appendix 2.2: GRADE evidence profile of the included studies. Risk of bias domain was downgraded as serious, owing to the high risk of the included studies. Other consideration (publication bias) was not assessed

**Table:**

Certainty assessment							No. of patients		Effect		Certainty	Importance
No of studies	Study design	Rick of bias	Inconsistency	Indirectness	Imprecision	Other consideration	Micro-invasive treatments	Non-invasive treatments	Relative (95% CI)	Absolute (95% CI)		
18	Randomizer trials	serious	Not serious	Not serious	Not serious	none	151/821 (18.4%)	354/819 (43.2%)	OR 0.23 (0.18–0.30)	283 fewer per 1000 patients (from 312–246)	⊕ ⊕ ⊕ ○ Moderate	
11	Randomizer trials	serious	Not serious	Not serious	Not serious	none	72/535 (13.5%)	213/538 (39.6%)	OR:0.21 (0.15–0.29)	275 fewer per 1000 patients (from 306–236)	⊕ ⊕ ⊕ ○ Moderate	
7	Randomizer trials	serious	Not serious	Not serious	Not serious	none	79/286 (27.6%)	141/281 (50.2%)	OR:0.27 (0.18–0.42)	288 fewer per 1000 patients (from 348–205)	⊕ ⊕ ⊕ ○ Moderate	

## Appendix 3: PRISMA 2009 Checklist

**Table:**

Section/topic	#	Checklist item	Reported on page #
*Title*			
Title	1	Identify the report as a systematic review, meta-analysis, or both	1
*Abstract*			
Structured summary	2	Provide a structured summary including, as applicable: background; objectives; data sources; study eligibility criteria, participants, and interventions; study appraisal and synthesis methods; results; limitations; conclusions and implications of key findings; systematic review registration number	1–2
*Introduction*			
Rationale	3	Describe the rationale for the review in the context of what is already known	2–4
Objectives	4	Provide an explicit statement of questions being addressed with reference to participants, interventions, comparisons, outcomes, and study design (PICOS)	4
*Methods*			
Protocol and registration	5	Indicate if a review protocol exists, if and where it can be accessed (e.g., Web address), and, if available, provide registration information including registration number	–
Eligibility criteria	6	Specify study characteristics (e.g., PICOS, length of follow-up) and report characteristics (e.g., years considered, language, publication status) used as criteria for eligibility, giving rationale	4–5
Information sources	7	Describe all information sources (e.g., databases with dates of coverage, contact with study authors to identify additional studies) in the search and date last searched	5–6
Search	8	Present full electronic search strategy for at least one database, including any limits used, such that it could be repeated	5–6
Study selection	9	State the process for selecting studies (i.e., screening, eligibility, included in systematic review, and, if applicable, included in the meta-analysis)	5–6
Data collection process	10	Describe method of data extraction from reports (e.g., piloted forms, independently, in duplicate) and any processes for obtaining and confirming data from investigators	6
Data items	11	List and define all variables for which data were sought (e.g., PICOS, funding sources) and any assumptions and simplifications made	6
Risk of bias in individual studies	12	Describe methods used for assessing risk of bias of individual studies (including specification of whether this was done at the study or outcome level), and how this information is to be used in any data synthesis	6
Summary measures	13	State the principal summary measures (e.g., risk ratio, difference in means)	7
Synthesis of results	14	Describe the methods of handling data and combining results of studies, if done, including measures of consistency (e.g., I^2^) for each meta-analysis	6–7

Section/topic	#	Checklist item	Reported on page #
Risk of bias across studies	15	Specify any assessment of risk of bias that may affect the cumulative evidence (e.g., publication bias, selective reporting within studies)	7
Additional analyses	16	Describe methods of additional analyses (e.g., sensitivity or subgroup analyses, meta-regression), if done, indicating which were pre-specified	7–8
*Results*			
Study selection	17	Give numbers of studies screened, assessed for eligibility, and included in the review, with reasons for exclusions at each stage, ideally with a flow diagram	8
Study characteristics	18	For each study, present characteristics for which data were extracted (e.g., study size, PICOS, follow-up period) and provide the citations	8–9
Risk of bias within studies	19	Present data on risk of bias of each study and, if available, any outcome level assessment (see item 12)	9
Results of individual studies	20	For all outcomes considered (benefits or harms), present, for each study: (a) simple summary data for each intervention group (b) effect estimates and confidence intervals, ideally with a forest plot	10–11
Synthesis of results	21	Present results of each meta-analysis done, including confidence intervals and measures of consistency	10–12
Risk of bias across studies	22	Present results of any assessment of risk of bias across studies (see Item 15)	12
Additional analysis	23	Give results of additional analyses, if done (e.g., sensitivity or subgroup analyses, meta-regression [see Item 16])	10–12
*Discussion*			
Summary of evidence	24	Summarize the main findings including the strength of evidence for each main outcome; consider their relevance to key groups (e.g., healthcare providers, users, and policy makers)	12–15
Limitations	25	Discuss limitations at study and outcome level (e.g., risk of bias), and at review-level (e.g., incomplete retrieval of identified research, reporting bias)	15–16
Conclusions	26	Provide a general interpretation of the results in the context of other evidence, and implications for future research	16
*Funding*			
Funding	27	Describe sources of funding for the systematic review and other support (e.g., supply of data); role of funders for the systematic review	17

From: Moher et al. [36]

For more information, visit: www.prisma-statement.org

## Abbreviations

OR: Odds ratio
RCTs: Randomised controlled trials
